# A pilot study of scleral thickness in central serous chorioretinopathy using anterior segment optical coherence tomography

**DOI:** 10.1038/s41598-021-85229-y

**Published:** 2021-03-12

**Authors:** Yun Ji Lee, Yeon Jeong Lee, Jae Yeon Lee, Suhwan Lee

**Affiliations:** 1 Department of Ophthalmology, Kangwon National University Hospital, Kangwon National University Graduate School of Medicine, Chuncheon, 24289 South Korea; 2 Hanvit Eye Center, Mokpo, 58652 South Korea

**Keywords:** Retinal diseases, Scleral diseases, Vision disorders

## Abstract

The aim of this study is to compare the scleral thickness of central serous chorioretinopathy (CSC) eyes with controls using anterior segment optical coherence tomography (AS OCT). This prospective case control study included 15 patients (15 eyes) with CSC and 15 age and gender matched healthy subjects. All subjects underwent spectral domain OCT with enhanced depth imaging and swept source AS OCT of temporal sclera. We investigated difference in scleral thickness between the two groups and relationship between choroidal and scleral thickness. Among the 15 eyes in the study group, 1 eye had acute CSC, 4 had recurrent CSC, 7 had inactive CSC, and 3 had chronic CSC. There was no significant difference in terms of age, gender, axial length and spherical equivalent between the two groups. The choroidal and scleral thickness of the study group were significantly greater than those of the control group (*P* < 0.001, *P* = 0.034). Choroidal thickness was positively correlated with scleral thickness (*P* = 0.031). A thick sclera along with a thick choroid were demonstrated in CSC eyes using AS OCT. Scleral characteristics might be involved in the pathogenesis of CSC by affecting outflow resistance of venous drainage in choroidal circulation.

## Introduction

Central serous chorioretinopathy (CSC) is a disease characterized by a localized exudative detachment of the neurosensory retina and retinal pigment epithelium (RPE)^1^. While the underlying pathophysiology of CSC remains unclear, choroidal vascular disturbance demonstrated on indocyanine green angiography (ICGA)—for instance, increased permeability of the choriocapillaris, vascular congestion, and venous dilation—is thought to be a principal mechanism behind the detachment of the neurosensory retina and RPE^2,3^. Recent studies using enhanced-depth imaging (EDI) optical coherence tomography (OCT) provided additional evidence supporting the role of the choroid in CSC^4^. Compared with healthy eyes, CSC eyes have increased subfoveal choroidal thickness (SFCT) (that is, pachychoroid) and dilated choroidal vessels (that is, pachyvessels)^4,5^.

As the choroid of the eye is primarily a vascular structure lacking in autoregulation, impairment in drainage of choroidal flow could cause congestion of the choroidal venous system, leading to pachychoroid and choroidal hyperpermeability, as seen in uveal effusion syndrome (UES)^6,7^. The pathogenesis of UES has been explained as outflow obstruction due to decreased transscleral outflow and compression of the vortex vein by an abnormal sclera (i.e., a thick and rigid sclera)^7,8^. Although the severity of choroidal congestion and exudative retinal detachment in CSC differs from that in UES, both are common findings in these conditions^9^. However, little information is available regarding the characteristics of sclera in CSC eyes. In this study, we aimed to investigate the scleral thickness of CSC eyes using anterior segment (AS) OCT and compare the results with those of normal eyes.

## Results

We enrolled 15 eyes from 15 patients diagnosed as CSC as the study group, and 15 eyes from 15 age- and gender-matched subjects as the control group. Among the 15 eyes in the study group, 1 eye had acute CSC, 4 had recurrent CSC, 7 had inactive CSC, and 3 had chronic CSC. At the time of enrollment, 6 out of 15 eyes in the study had no prior treatment, while 9 eyes had received different treatments (Table 1). The study group comprised 11 men and 4 women with a mean age of 50.27 ± 14.42 years (range 28–76 years), and the control group comprised 13 men and 2 women with a mean age of 54.73 ± 11.82 years (range 30–72 years). The two groups did not differ significantly in terms of age, gender, AL and spherical equivalent (*P* = 0.361, *P* = 0.651, *P* = 0.125, *P* = 0.878, respectively) (Table 2). The main systemic disease involving taking of medication in both the groups was cardiovascular diseases, including hypertension and coronary heart diseases, and no significant difference was noted between the two groups in terms of their presence (*P* = 0.143).

**Table 1:** Characteristics of study groups related to CSC.

Patient no.	Age	Gender	Predisposing systemic factors	CSC subtype	No of leaks in FA during active phase	Treatment history
1	50	M	None	Recurrent	Unifocal	MRA
2	28	M	None	Inactive	Unifocal	No treatment
3	52	F	None	Inactive	Unifocal	No treatment
4	46	F	None	Inactive	Unifocal	Anti-VEGF
5	73	M	CHD	Chronic	Multifocal	No treatment
6	60	M	HTN, CHD	Inactive	Multifocal	Anti-VEGF
7	56	M	CHD	Recurrent	Unifocal	MRA
8	36	M	None	Recurrent	Unifocal	Focal laser
9	44	M	None	Recurrent	Multifocal	Focal laser, anti-VEGF
10	67	M	HTN	Chronic	Multifocal	Anti-VEGF
11	56	M	None	Inactive	Unifocal	Anti-VEGF
12	45	M	None	Inactive	Unifocal	No treatment
13	76	M	HTN	Chronic	Multifocal	Anti-VEGF
14	33	F	Pregnancy	Acute	NA^a^	No treatment
15	35	F	Pregnancy	Inactive	NA^a^	No treatment

*CSC* central serous chorioretinopathy, *MRA* mineralocorticoid receptor antagonist, *VEGF* vascular endothelial growth factor, *CHD* coronary heart disease, *HTN* hypertension. ^a^FA was not performed because of pregnancy.

**Table 2:** Comparison of demographic and ocular characteristics between study and control groups.

	Study group	Control group	*P* value
Male/female, no	11/4	13/2	0.651
Age, mean (SD), years	50.27 (14.42)	54.73 (11.82)	0.361
Axial length, mean (SD), mm	23.34 (0.96)	23.78 (0.52)	0.125
Spherical equivalent, median (IQR), diopter	− 0.38 (− 1.63 to 0.75)	− 0.75 (− 1.25 to 0.50)	0.878
Choroidal thickness, median (IQR), µm	443.0 (389.0–474.0) (range 319.0–748.0)	294.0 (281.0–330.0) (range, 212.0–333.0)	< 0.001
Scleral thickness, median (IQR), µm	418.5 (370.5–461.5) (range 343.0–597.0)	374.0 (352.5–405.5) (range, 309.0–486.0)	0.034

**Figure 1:**
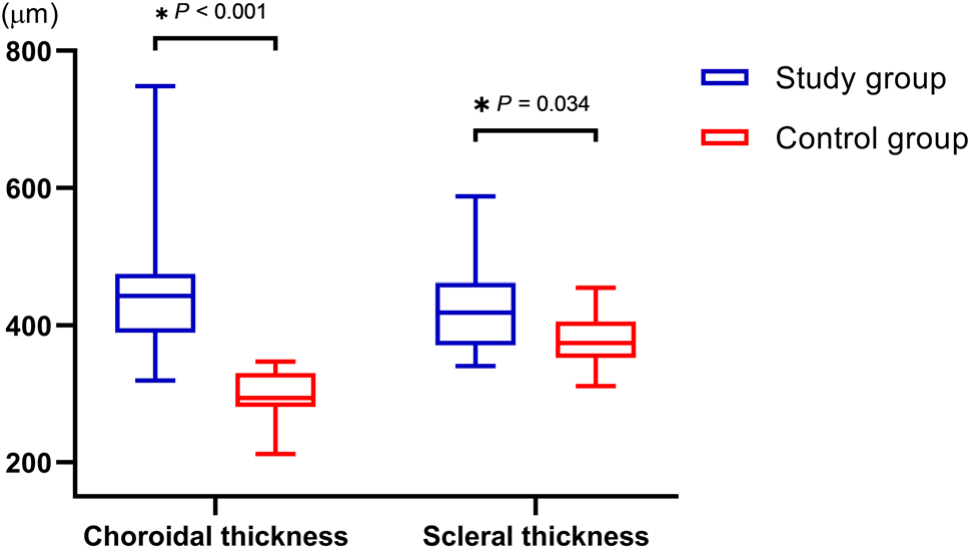
Box plot images showing the difference in choroidal thickness and scleral thickness between study and control groups. Upper and lower bars represent maximum and minimum values, respectively.

**Figure 2:**
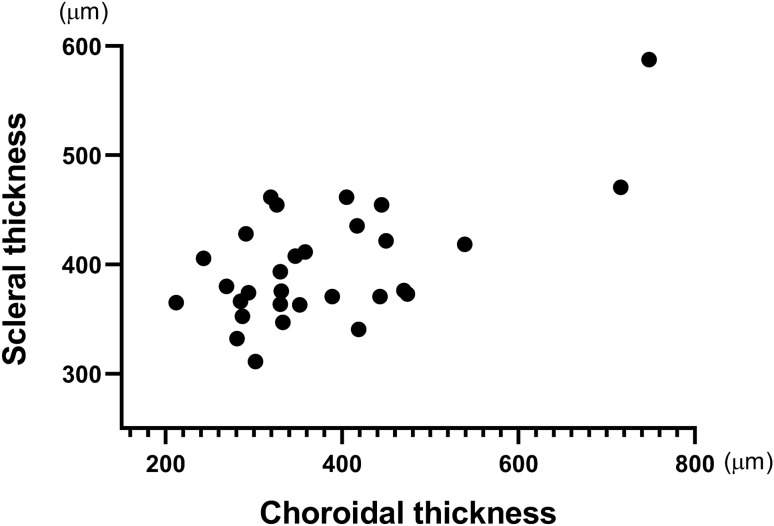
Relationship between choroidal thickness and scleral thickness in all participants.

**Figure 3:**
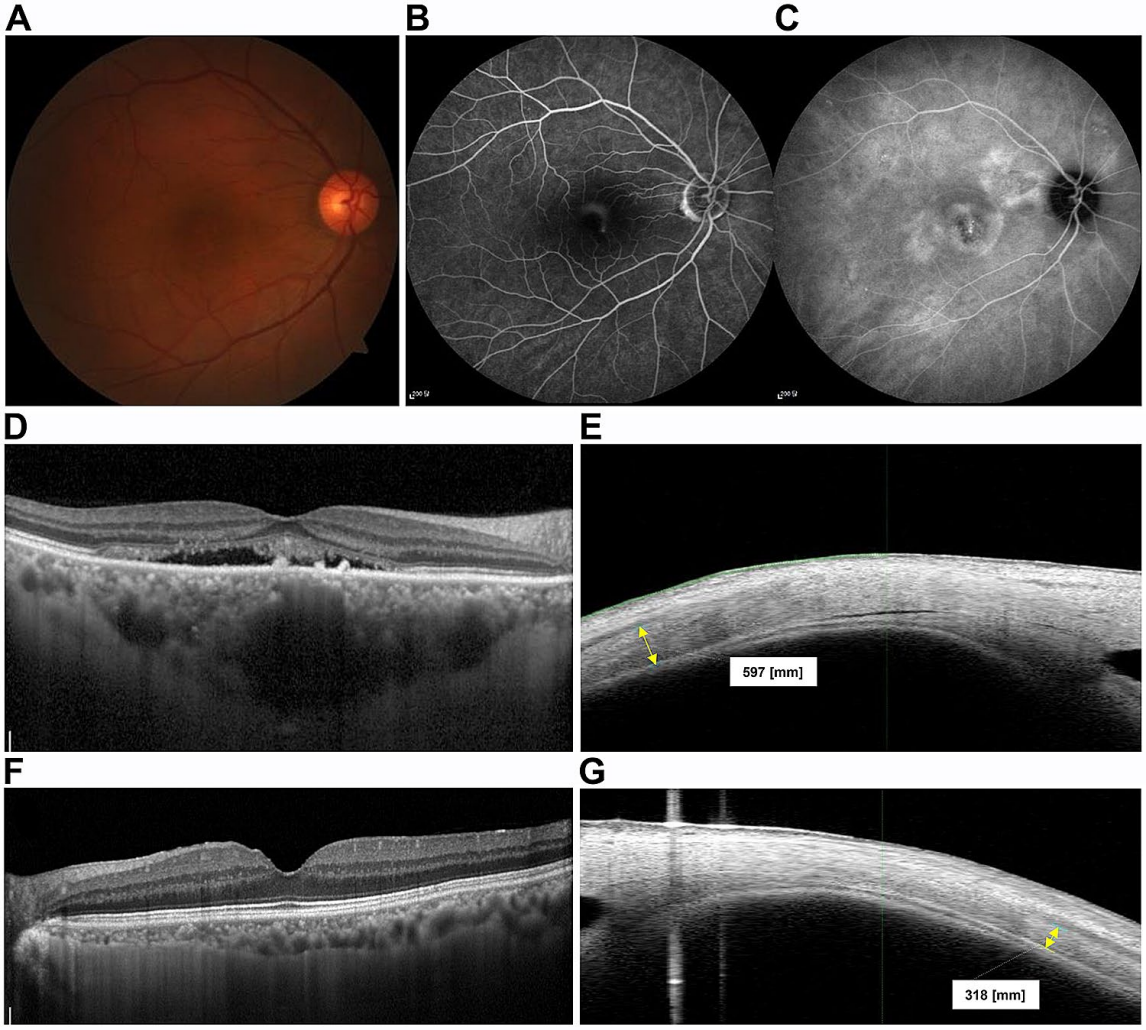
Representative cases of CSC eyes (**A**–**E**) and normal eyes (**F**,**G**). Color fundus photographs of CSC eyes show serous macular detachment (**A**) and fluorescein angiography shows typical smoke stack pattern of leakage (**B**). Late phase image of indocyanine angiography shows multiple areas of choroidal hyperpermeability. We observed very thick choroid in enhanced-depth imaging (EDI) of optical coherence tomography (OCT) and relatively thick sclera in swept source anterior segment (AS) OCT in CSC eyes (**D**,**E**), compared with those of normal eyes (**F**,**G**).

The median SFCT in the study and control groups was 443.0 µm (interquartile range 389.0–474.0 µm; range 319.0–748.0 µm) and 294.0 µm (interquartile range 281.0–330.0 µm; range 212.0–333.0 µm), respectively (Table 2; Fig. 1). The median sub-LR scleral thickness measured in the study group was 418.5 µm (interquartile range 370.5–461.5 µm; range 343.0–597.0 µm) while that in the control group was 374.0 µm (interquartile range 352.5–405.5 µm; range 309.0–486.0 µm) (Table 2; Fig. 1). We observed a statistically significant difference in both the SFCT and the sub-LR scleral thickness of the two groups (*P* < 0.001, *P* = 0.034, respectively). Correlation analysis showed a moderate positive linear relationship between SFCT and the sub-LR scleral thickness (*r* = 0.394, *P* = 0.031) (Fig. 2). The intraclass correlation coefficient (ICC) values for the intra-observer and inter-observer reliability for the measurement of the scleral thickness demonstrated high levels of repeatability (ICC = 0.916, *P* < 0.001, ICC = 0.950, *P* < 0.001, respectively). Figure 3 displays these representative cases.

## Discussion

In this study, we examined the scleral thickness of CSC eyes to investigate the possible role of the sclera in the pathogenesis of pachychoroid. We found that both the scleral and the choroidal thickness of CSC eyes were significantly greater than those of healthy eyes. Additionally, choroidal thickness and scleral thickness were positively correlated. These results are consistent with those of Imanaga et al., who first reported thick sclera in eyes with CSC^10^. They measured scleral thickness in four quadrants using AS OCT with more cases, providing further strong evidence for the hypothesis of thick sclera in CSC eyes.

Venous drainage of the choroidal circulation is thought to occur primarily through the four vortex veins. Pang et al., using ultra-wide field ICGA, observed dilated choroidal vessels in CSC eyes and identified these dilated vessels as macular branches of the vortex vein, noting that the entire course of these vessel from the distal ends to ampullas of the vortex veins were markedly dilated, suggesting outflow congestion of the draining vortex vein^11^. The large vessels located in the outer choroidal layer are known to be the major contributors to the increase in the choroidal thickness seen with EDI OCT in CSC^5,12,13^. Morphologic features of these large vessels were visualized using en face swept source OCT, and corresponded with the macular branch of the vortex veins observed in ICGA^14–16^. Based on these results, the engorgement of the macular branch of the vortex vein associated with outflow congestion is thought to be a major contributing factor to increased choroidal thickness in CSC.

Because the vortex vein emerges from the eyeball only through the narrow scleral tunnel, its outflow pathway could be affected by the characteristics of the sclera, which is a relatively rigid structure in the eye^17^. Eyes with thick sclerae may have narrower and longer scleral tunnels, which would increase the outflow resistance of the vortex vein, causing a bottleneck phenomenon. The primary pathogenic mechanism behind the development of UES is believed to be a thickened and rigid sclera, which impedes transscleral outflow and causes congestion of the vortex vein^9,18^. Successful results of surgical procedures such as subscleral sclerectomy and vortex vein decompression in UES have further strengthened the evidence of the abnormal sclera as the primary pathology^7,19^. Interestingly, Venkatesh et al. reported a similar case in a CSC patient^20^. In eyes with chronic CSC complicated by exudative retinal detachment, they performed partial thickness scleral resection with mitomycin C, which resulted in improved visual acuity and resolution of subretinal fluid. In contrast, several reports showed temporary thickening of the SFCT after scleral buckling^21–23^. Iwase et al. suggested that in such cases venous drainage obstruction induced by the compression force of the scleral buckle leads to choroidal thickening^22^. These cases are believed to be good examples reflecting the relationship among the sclera, the vortex vein, and choroidal congestion.

Various studies have implicated several risk factors associated with CSC development. Recently, two large case–control studies simultaneously reported hyperopia as an independent risk factor for CSC^24,25^. In both studies, consistent with the study by Manayath et al., myopia was found to protect from CSC^26^. Hyperopia |usually indicates that the eyes have a short AL; the AL of unilateral idiopathic CSC and fellow eyes was also reported to be shorter than that of control eyes^27^. Scleral thickness and choroidal thickness are well known to decrease with axial elongation of the globe^28,29^. Conversely, in small hyperopic eyes, the sclera is relatively thick. Therefore, we postulated that the underlying pathology of small hyperopic eyes as a risk factor for CSC might be associated with relatively thick sclerae, which would have increased choroidal circulation resistance outflow by the same mechanism as that in the case of UES which occurs primarily in short eyes.

Based on previous studies and our findings, we concluded that the pathogenesis of CSC might be characterized by an imbalance between the inflow and outflow of choroidal circulation. In other words, in eyes with decreased outflow capacity, increased choroidal inflow would cause choroidal congestion, leading to serous RPE and retinal detachment. In contrast, eyes with adequate outflow capacity could readily dispose of increased choroidal inflow without causing choroidal congestion. Scleral characteristics and their relationship with the vortex vein may be important factors affecting the outflow capacity. Tittle et al. reported a significant increase in choroidal blood flow after isometric exercise in chronic CSC patients compared with that in healthy subjects^30^. Compared with normal controls, patients with CSC also exhibited dynamic choroidal thickening in response to increased perfusion pressure induced by postural changes^31^. These results in CSC eyes might represent impairment of choroidal outflow preventing the ready disposal of rapidly increasing choroidal inflow.

**Figure 4:**
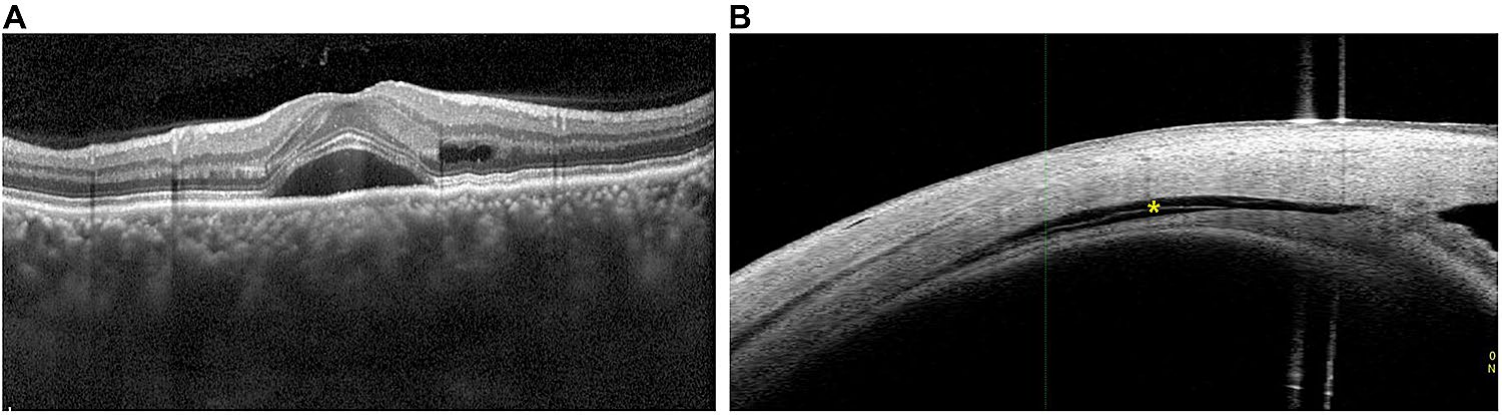
We observed anterior choroidal effusion (asterisk, **B**) in a CSC eye with pachychoroid (**A**).

This study has the following limitations. First, it involved a small sample size, which limited the statistical strength of the analysis. Second, because there is no anatomical reference point in the measurement of scleral thickness (as, for instance, the foveolar in the case of choroidal thickness), it was difficult to compare the thickness of the sclera at any particular point. Moreover, we only measured the temporal quadrant because it was difficult to expose the sclera for accurate measurements in other quadrants. However, we cannot rule out the possibility that confounding factors specific to temporal area may have affected our results. Nevertheless, the findings by Imanaga et al. suggest that these effects are minimal^10^. To Third, since it is hard to capture the entire sclera under the posterior pole using currently available imaging modalities, we measured scleral thickness at the insertion site of the lateral rectus muscle using AS OCT. However, in UES, the entire sclera from its anterior to its posterior area is thickened, and CSC is suggested to be an extensive disease not limited to the posterior pole^7,11^. One of our cases did indeed show anterior choroidal effusion, which was demonstrated by AS OCT (Fig. 4). The vortex vein is also located at the periphery, not the posterior pole^17^; it is therefore an unsuitable location for observing the difference between CSC eyes and normal eyes. By investigating the sclera in CSC, we have proposed another aspect for consideration regarding the pathogenesis of CSC. Further study with a larger sample size and imaging modalities that facilitate more accurate measurement of scleral thickness will allow for confirmation of our results.

In conclusion, in eyes with CSC, both the sclera and the choroid were thicker than those in normal eyes. Scleral characteristics, by affecting outflow resistance, may be another contributing factor in the development of CSC. Therefore, the balance between inflow and outflow of choroidal circulation should be considered and novel treatment strategies aimed at relieving outflow obstruction are indicated in the management of CSC.

## Methods

We performed this prospective, cross-sectional, case–control study at Kangwon National University Hospital, Chuncheon, Korea between October 2019 and November 2019. The institutional review board of Kangwon National University Hospital approved the study, which adhered to the tenets of the Declaration of Helsinki. All patients gave their written informed consent prior to their enrollment in the study. We consecutively enrolled patients diagnosed with CSC as the study group, and formed the control group from age- and gender-matched healthy subjects. We based diagnosis of CSC on ocular examination including spectral-domain (SD) OCT and fluorescein angiography (FA) results. CSC is classified in accordance with the suggestion of Daruich et al., and all subtypes of CSC were included in this study^1^. We excluded patients with (1) a choroidal thickness < 300 µm; (2) an axial length > 25 mm; (3) any other retinal abnormalities, including diabetic retinopathy, retinal vein occlusion, choroidal neovascularization, and posterior uveitis; (4) previous vitreoretinal surgery and trauma; (5) a history of taking medications that could induce CSC-like retinopathy, such as mitogen-activated protein kinase inhibitors^32^; (6) poor-quality images that could not be evaluated because of low resolution.

All participants underwent a comprehensive ophthalmologic examination, including measurement of best-corrected visual acuity, intraocular pressure, slit-lamp biomicroscopy, and dilated fundus examination. We measured axial length (AL) with a partial coherence laser interferometer (IOL master 500; Carl Zeiss Meditec AG, Jena, Germany), and determined choroidal thickness from images obtained using the EDI mode of a Spectralis OCT device (Heidelberg Engineering Inc, Heidelberg, Germany), analyzing them with the associated software (Version 6.0). We obtained the horizontal and vertical sections, comprising 100 averaged scans passing directly through the center of the fovea, in a 5° × 30° rectangle centered on the macula. We measured the SFCT from the outer portion of the hyperreflective line corresponding to the RPE to the inner surface of the choroid/scleral boundary. Two independent graders measured the SFCT and we used the average measurements in statistical analysis.

### Measurement of scleral thickness

**Figure 5:**
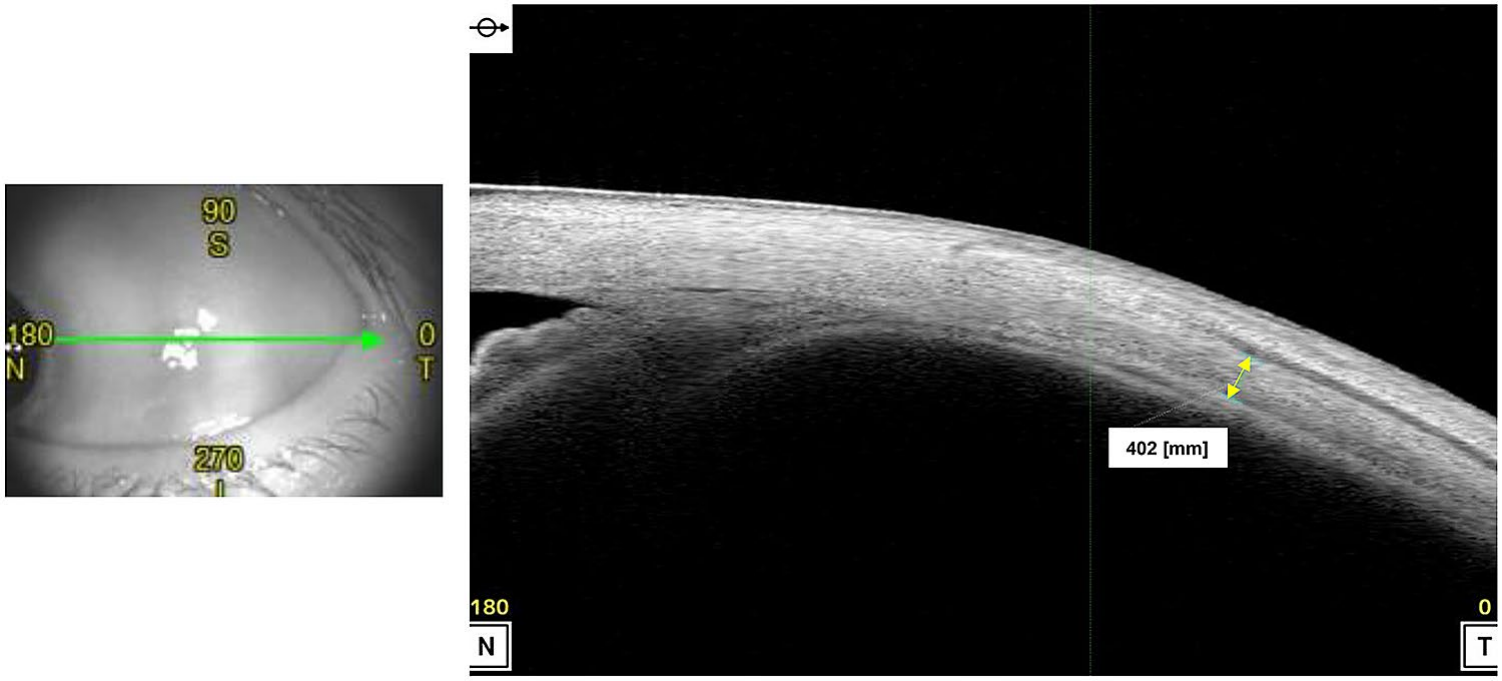
Method of obtaining image of temporal sclera and measuring scleral thickness using anterior segment optical coherence tomography.

We imaged the eyes of all participants with a swept source AS OCT (CASIA2; Tomey Corporation, Nagoya, Japan). We captured images of temporal sclera during 45° nasal gaze position using ‘bleb wide’ mode (Fig. 5). Raster scans of ‘bleb wide’ mode comprised 256 high-quality cross-sectional images of horizontal and vertical lines over the sclera with 400 A-scans per line sampling. Of all scanned images, we selected one best-quality image of the horizontal section perpendicular to the corneal limbus for analysis. We manually measured scleral thickness at the insertion site of the lateral rectus (LR) using built-in 2D Analysis software (Version 3E.2), measuring perpendicularly from the LR muscle/scleral interface to the scleral/choroidal boundary. Two examiners (YJ Lee and JY Lee) independently performed the measurements of sub-LR scleral thickness and we used the average measurements in statistical analysis.

### Statistical analysis

We used SPSS version 22.0 software (SPSS, Inc., Chicago, IL, USA) to perform the statistical analysis, and considered *P* < 0.05 to be statistically significant. We performed the Shapiro–Wilk test on various continuous variables for the assessment of Gaussian distribution. We present descriptive data as mean (standard deviation) or median (interquartile range), and we used the Student *t* test or Mann–Whitney *U* test as statistical methods of comparing variables, as determined based on the above results. We performed Spearman’s correlation analysis to examine the relationship between choroidal thickness and scleral thickness. We expressed the inter-observer reliability of the scleral thickness measured by the two observers as the ICC.
