# Author Correction: A pilot study of scleral thickness in central serous chorioretinopathy using anterior segment optical coherence tomography

**DOI:** 10.1038/s41598-021-93183-y

**Published:** 2021-07-06

**Authors:** Yun Ji Lee, Yeon Jeong Lee, Jae Yeon Lee, Suhwan Lee

**Affiliations:** 1 Department of Ophthalmology, Kangwon National University Hospital, Kangwon National University Graduate School of Medicine, Chuncheon, 24289 South Korea; 2 Hanvit Eye Center, Mokpo, 58652 South Korea

Correction to: *Scientific Reports* https://doi.org/10.1038/s41598-021-85229-y, published online 12 March 2021

The original version of Article contained errors.

Reference 10 was omitted and is listed below:

 Imanaga, N. *et al.* Scleral Thickness in Central Serous Chorioretinopathy. *Ophthalmology. Retina* **5**, 285-291 (2021).

As a result, References 11-32 were incorrectly listed as References 10-31.

Consequently, the text in the Discussion,

“We found that both the scleral and the choroidal thickness of CSC eyes were significantly greater than those of healthy eyes. Additionally, choroidal thickness and scleral thickness were positively correlated”

now reads:

“We found that both the scleral and the choroidal thickness of CSC eyes were significantly greater than those of healthy eyes. Additionally, choroidal thickness and scleral thickness were positively correlated. These results are consistent with those of Imanaga et al., who first reported thick sclera in eyes with CSC^10^. They measured scleral thickness in four quadrants using AS OCT with more cases, providing further strong evidence for the hypothesis of thick sclera in CSC eyes.”

And the text,

“However, we cannot rule out the possibility that confounding factors specific to temporal area may have affected our results.”

now reads:

“However, we cannot rule out the possibility that confounding factors specific to temporal area may have affected our results. Nevertheless, the findings by Imanaga et al. suggest that these effects are minimal^10^.”

The original Article has been corrected.

